# Between-Subject Variability in the Breaking Continuous Flash Suppression Paradigm: Potential Causes, Consequences, and Solutions

**DOI:** 10.3389/fpsyg.2017.00437

**Published:** 2017-03-27

**Authors:** Surya Gayet, Timo Stein

**Affiliations:** ^1^Department of Experimental Psychology, Utrecht UniveristyUtrecht, Netherlands; ^2^Center for Mind/Brain Sciences, University of TrentoRovereto, Italy; ^3^Amsterdam Brain and Cognition, University of AmsterdamAmsterdam, Netherlands

**Keywords:** consciousness, visual awareness, continuous flash suppression, binocular rivalry, individual differences, response times, normalization, assumption of normality

## Abstract

A recent focus in the field of consciousness research involves investigating the propensity of initially non-conscious visual information to gain access to consciousness. A critical tool for measuring conscious access is the so-called breaking continuous flash suppression paradigm (b-CFS). In this paradigm, a high contrast dynamic pattern is presented to one eye, thereby temporarily suppressing a target stimulus that is presented to the other eye. The time it takes for observers to report (e.g., the location of) the initially suppressed stimulus provides a measure of conscious access. Typical observations in b-CFS studies include the finding that upright faces are released from suppression faster than inverted faces, and the finding that stimuli that match the current content of visual working memory are released from suppression faster than mismatching stimuli. Interestingly, the extent to which observers exhibit these effects varies extensively (in the range of hundreds of milliseconds). By re-analyzing existing datasets and a new dataset we establish that the difference in RTs between conditions in b-CFS tasks (i.e., the effect of interest) is highly correlated with participants' overall suppression durations, and with their trial-to-trial variability in RTs. We advocate the usage of a simple latency- normalization method, which (1) removes the between-subject variability in suppression duration from the effect of interest, while (2) providing distributions of RT differences that are better suited for parametric testing. We next compare this latency-normalization method to two other transformations that are widely applied on within-subject RT data (z-transformations and log-transformations). Finally, we tentatively discuss how trial-to-trial variability and overall suppression duration might relate to prolonged phases of shallow suppression that are more prone to modulations of conscious access.

## Introduction

Consciousness researchers have many psychophysical tools at their disposal to render visual input invisible to the observer (for reviews, see Kim and Blake, 2005; Breitmeyer, 2015; Overgaard, 2015). One method in particular, however, has been increasingly dominant in the recent consciousness literature. Continuous flash suppression (CFS; Tsuchiya and Koch, 2005) consists of presenting a high contrast dynamic masking stimulus to one eye, thereby causing prolonged suppression of a lower contrast static target stimulus presented to the other eye. In 2015 alone, more than 40 studies have been published that use CFS. In particular, many studies use the time it takes for an initially suppressed target stimulus to overcome CFS as a measure of conscious access. This method is referred to as “breaking continuous flash suppression” (b-CFS; Jiang et al., 2007; Stein et al., 2011a; for a review, see Gayet et al., 2014).

Recent findings using this method have sparked the debate about the extent to which visual input is processed in the absence of consciousness (e.g., Hassin, 2013; Gayet et al., 2014; Yang et al., 2014; Hesselmann and Moors, 2015). Response times (RTs) in b-CFS experiments are lower than in traditional detection tasks, where information is not interocularly suppressed. Simultaneously, raw RT differences between experimental conditions are typically larger (in absolute time) than those typically observed with traditional detection tasks (e.g., comparing Stein et al., 2011a, with Lewis and Ellis, 2003). Eyeballing individual participants' b-CFS data (e.g., in Jiang et al., 2007; Wang et al., 2012) reveals an interesting data pattern: participants with slower overall RTs across conditions typically exhibit a larger RT difference between conditions. A number of recent b-CFS studies explicitly reported this correlation between overall RT and raw RT difference between conditions (e.g., Gayet et al., 2016a,b). Similarly, it has been demonstrated that artificially lengthening the RTs (e.g., by reducing the contrast of the target or by using more potent masks) induces larger raw RT differences (Stein et al., 2011a). As such, the relatively slow RTs in the b-CFS paradigm might play a role in the paradigm's potency for uncovering (small) differences in processing strength between stimulus conditions, which would drown in noise using traditional methods. Another interesting observation is that the trial-to-trial variability in RTs in b-CFS experiments is higher than in traditional detection tasks where information is not interocularly suppressed (i.e., within-participant variance is typically much larger in b-CFS conditions than in control conditions involving no interocular competition). Greater variability in detection times implies that observers have greater uncertainty regarding the appearance of a target stimulus. This might also play a role in the b-CFS paradigm's sensitivity for detecting differences between conditions (for a discussion, see Stein et al., 2011a). Considering that the b-CFS paradigm is widely employed to answer important theoretical and philosophical questions on consciousness, it is important to better understand the mechanisms that play a role in the potency of the b-CFS method in uncovering differences in visual processing between experimental conditions.

The purpose of this article is 3-fold. First, we empirically establish the abovementioned relationships between overall RTs, trial-to-trial variability and raw RT differences within the b-CFS paradigm. For this, we use two of the most replicated findings of the b-CFS literature: face inversion and VWM boost. For those effects, we demonstrate that a participant's raw RT difference between two experimental conditions is strongly correlated with (A) the participant's overall RT across conditions and (B) the participant's trial-to-trial variability in RTs. As it is well established that overall RTs correlate with RT variability (Wagenmakers and Brown, 2007), a correlation of both metrics with RT differences is expected. Second, we suggest that this observation is of particular relevance in the b-CFS paradigm, where between-subject variability in RTs stems from both interindividual differences in interocular suppression durations, as well as interindividual differences in response speed after the interocular conflict is resolved. Following this consideration, we provide a clear-cut solution for handling data in which such a correlation is observed. The proposed latency-normalization procedure, which removes between-subject variability of-no-interest, has been used in only a few previous b-CFS studies. Here, we show for the first time that (1) this latency-normalization procedure yields RT differences that more closely approximate a normal distribution, and (2) we make the case that it provides increased sensitivity for detecting differences between experimental conditions. Third, we tentatively propose that the correlation between overall RTs, trial-to-trial variability in RTs, and raw RT differences between conditions could reflect the role of perceptual uncertainty in modulating RTs in the b-CFS paradigm. Finally, we discuss how this putative role of perceptual uncertainty could allow for reconciling contradictory and surprising findings that have been observed in recent b-CFS studies.

For these purposes, we retrieved data from three existing b-CFS datasets that we deemed most representative of the b-CFS paradigm. Following Gayet et al. (2014) we distinguished between two types of manipulations in the b-CFS paradigm. The first type of manipulations in b-CFS paradigms consists of manipulations of stimulus *content*, in which two different stimuli are compared in their propensity to reach conscious access (e.g., faces vs. houses). Arguably, the most reliable manipulation of stimulus content in the b-CFS literature is the finding that upright faces are released from interocular suppression faster than inverted faces (e.g., Jiang et al., 2007; Stein et al., 2011a). This face inversion effect is of particular interest because it involves the comparison of stimuli with identical pixel values, differing only in their spatial orientation on the screen. This approach thus allows for ruling out many low-level stimulus confounds that could explain differences in suppression durations between image categories (Stein et al., 2012). The second type of manipulations in b-CFS paradigms consists of manipulations of stimulus *context*, in which conscious access of a single stimulus is assessed as a function of its relation with consciously accessible information (e.g., a target following a congruent vs. an incongruent cue). With this approach, by definition, stimuli in different conditions are identical, such that low-level stimulus contributions cannot account for any difference in suppression durations between conditions. The most reliable manipulation of stimulus context arguably consists of manipulating the concurrent content of visual working memory. Applying this manipulation revealed that visual input that matches concurrently memorized visual features (such as a color or shape) is released from interocular suppression faster than visual input that mismatches the concurrently memorized content (Gayet et al., 2013, 2016a; Pan et al., 2014; van Moorselaar et al., 2015, 2016; Gayet, 2016).

## Methods

### Datasets

For the present set of analyses, we used three b-CFS datasets, one from an experiment that has not yet been published, and two datasets were retrieved from published studies (Gayet et al., 2013; Gayet, 2016). The first dataset comprises of a b-CFS experiment in which RTs to upright faces were compared with RTs to inverted faces (see Appendix for a complete description of the Methods). For the sake of brevity, this dataset will be referred to as the “Face Inversion” experiment. It revealed that upright faces are released from interocular suppression faster than inverted faces. The second dataset comprises of a b-CFS experiment in which RTs to colored targets are compared between the case in which they match and the case in which they mismatch a color that is concurrently maintained in visual working memory for a subsequent recall task. Similarly, the third dataset comprises of a b-CFS experiment in which RTs to geometrical target shapes are compared between the case in which they match and the case in which they mismatch a geometrical shape that is concurrently maintained in visual working memory. These datasets will be referred to as the “Color” experiment and the “Shape” experiment respectively (see Gayet et al., 2013; Gayet, 2016, for a complete description of the Methods in these two experiments). In short, the Color and Shape Experiments revealed that identical visual stimuli are released from interocular suppression faster when they match a stimulus that is concurrently maintained in visual working memory (for a subsequent recall task). The left part of Table 1 provides general information on these three experiments, including the number of participants, the overall mean RT and the trial-to-trial variability (see the “metrics” paragraph below).

**Table 1:** **General information, and effect size and normality comparisons for all three data sets**.

**Manipulation**	**Latency/variability (SD)**	**RT difference**	**Effect (SD)**	***t*-value**	**Cohen's *d***	**BF_10_**	**W**
Face inversion		Raw	636 ms (573)	8.945	1.109	1 × 10^10^	0.857^*^
*N* = 65	RT_OVERALL_: 2069 ms (784)	Latency-normalized	26% (16)	13.415	1.664	2 × 10^17^	0.966
	SD_WITHIN_: 935 ms (500)	Z-transformed	0.52 (0.36)	11.581	1.436	2 × 10^14^	0.930^*^
		Log-transformed	0.10 (0.05)	15.912	1.974	6 × 10^20^	0.976
Color memory		Raw	230 ms (242)	5.628	0.951	7 × 10^3^	0.848^*^
*N* = 35	RT_OVERALL_: 1955 ms (1035)	Latency-normalized	12% (10)	6.461	1.092	7 × 10^4^	0.954
	SD_WITHIN_: 685 ms (429)	Z-transformed	0.22 (0.20)	6.526	1.103	9 × 10^4^	0.948
		Log-transformed	0.05 (0.05)	5.610	0.948	7 × 10^3^	0.947
Shape memory		Raw	105 ms (140)	3.353	0.769	12	0.774^*^
*N* = 19	RT_OVERALL_: 1819 ms (708)	Latency-normalized	11% (11)	4.418	1.014	100	0.951
	SD_WITHIN_: 600 ms (180)	Z-transformed	0.08 (0.12)	3.252	0.746	10	0.820^*^
		Log-transformed	0.02 (0.02)	4.238	0.972	68	0.950

* *Significant violation of normality according to the Shapiro-Wilk normality test*.

### Metrics

In order to investigate the relation between overall RTs, trial-to-trial RT variability and the RT difference between experimental conditions, we established the following metrics. Each Participant's overall RT (RT_OVERALL_) was defined as the average of the median RTs in each experimental condition that elicited a RT difference (i.e., upright and inverted, or matching and mismatching conditions, but also left and right eye, different target locations, and differently colored or shaped targets). Medians were used to account for the skewness in the distributions of raw RTs, and for including trials that yielded no response within the time window (reflecting the longest suppression durations). The trial-to-trial variability was computed as the average of the SDs of each experimental condition that elicited a RT difference, hence yielding a within-condition SD (or SD_WITHIN_), which only reflects RT variability that is not caused by the experimental manipulations. The raw RT difference was computed by subtracting each participants' median RT to upright faces (or matching targets) from the median RT to inverted faces (or mismatching targets).

### Analysis

We computed standard Pearson correlations, which were regarded as significant in case the *p*-value was below the threshold of α = 0.05 divided by the number of parallel comparisons, following the Bonferroni correction. In addition, we ran one-sided Bayesian correlation analyses, testing for the evidence or absence of a positive correlation (using JASP Team, 2016; Beta prior width of 1).

## Results I: breaking continuous flash suppression

### Overall RTS

**Figure 1:**
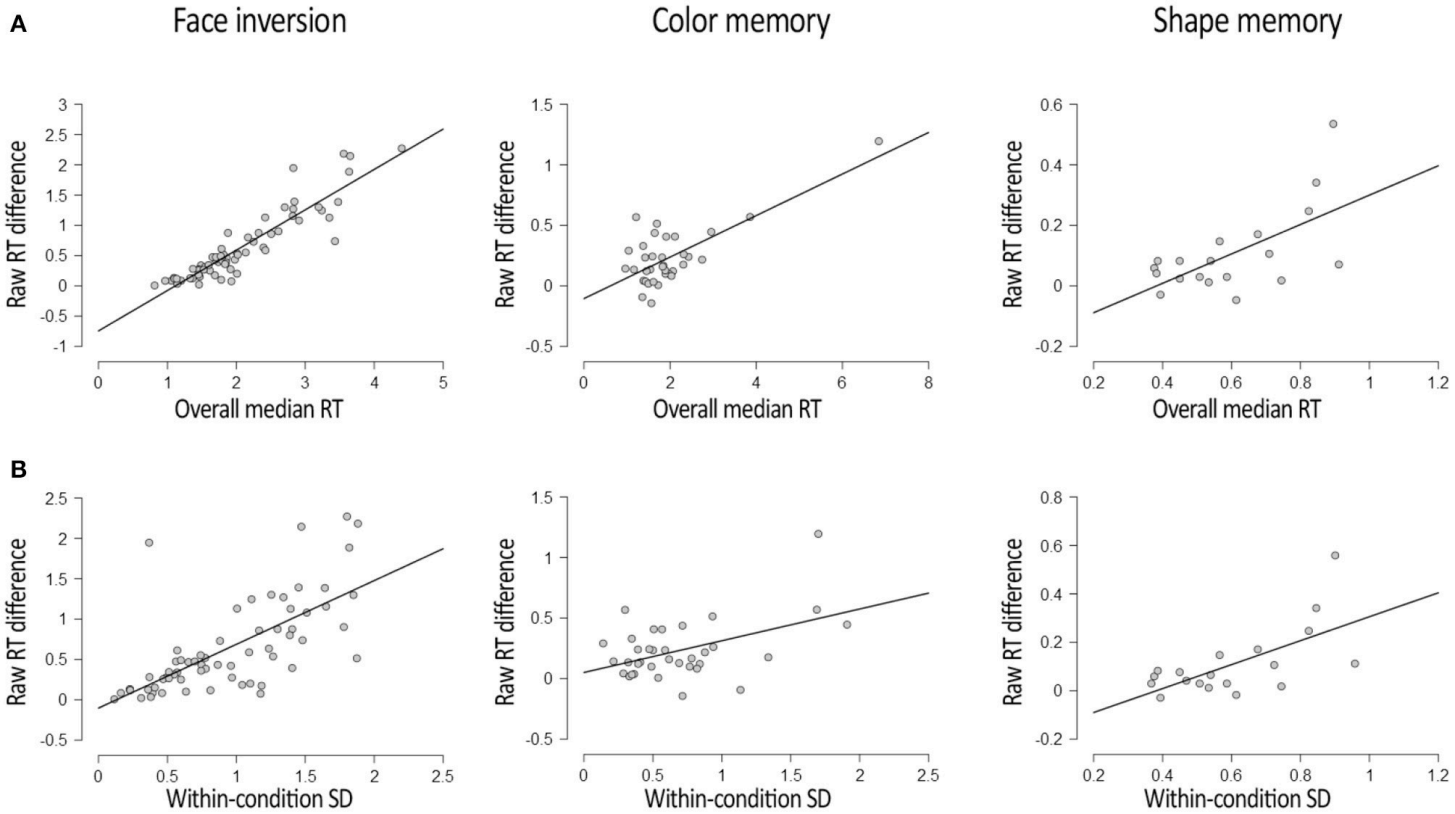
**Results of the main correlational analyses in all three datasets. (A)** Depicts the correlation between participants' overall RT (x-axis) and the RT difference between experimental conditions (y-axis) for all three datasets. **(B)** Depicts the correlation between the within-condition variability of RTs (x-axis) and the RT difference between experimental conditions (y-axis) for all three datasets. All correlations depicted here were significant after Bonferroni correction, with all *p*'s < 0.005. Plots were adapted from JASP Team (2016) output.

The results of the main correlational analyses are depicted in Figure 1A. In all three datasets reported here, participants' overall RTs correlated with the difference in raw RTs between experimental conditions. This was true for the Face Experiment, *R* = 0.912, *p* < 0.001, BF_+0_ = 7^*^10^71^, for the Color Experiment, *R* = 0.726, *p* < 0.001, BF_+0_ = 2^*^10^4^, and for the Shape Experiment, *R* = 0.687, *p* = 0.001, BF_+0_ = 38. This correlation has been reported in the b-CFS literature on a number of other occasions as well (e.g., Gayet et al., 2016a,b), and can also be observed when eyeballing individual participant's data in a number of other b-CFS studies (e.g., Jiang et al., 2007; Wang et al., 2012). Thus, this appears to be a common phenomenon in b-CFS data.

### A simple latency-normalization procedure

The present results show that, for different experimental manipulations in the b-CFS paradigm, participants with slower overall RTs also show a stronger RT difference between experimental conditions. In other words, it is possible that part of the between-subject variability in the RT difference between experimental conditions (i.e., the researcher's effect of interest) stems from between-subject variability in overall RTs (i.e., which is not related to the effect of interest). Consequently, when conducting statistical analyses to establish whether an effect of interest is present or absent, statistical power is reduced by between-subject variability that is unrelated to the effect of interest. A simple way to isolate the effect of interest from participants' overall response speed is to normalize the RT difference for each participant, such that it reflects a proportional (rather than absolute) difference in RTs caused by the experimental manipulation. This can be achieved by dividing the difference in RT between two conditions by the overall RT:

$$\begin{array}{l} \Delta RT_{\text{NORMALIZED}}\;=100*\;\frac{RT_{A}-\;RT_{B}}{RT_{OVERALL}} \end{array}$$

A similar approach has been proposed by Tsuchiya et al. (2009), by Stein (2012); Stein et al. (2011b, 2012), and by Gayet et al. (2016a); Gelbard-Sagiv et al. (2016).

In Table 1, frequentist and Bayesian test-statistics, as well as standardized effect sizes (Cohen's *d*) are provided for the raw RT difference and the normalized RT difference of all three datasets. This reveals that the latency-normalization procedure increased the sensitivity to detect differences between experimental conditions in all three datasets, as revealed by increased *t*-statistics and larger standardized effect sizes (Cohen's *d*) relative to the tests performed on the raw RT differences. This advantage of the normalization procedure was also observed in Gayet et al. (2016b; Supplementary Materials). Thus, although we do not provide statistical confirmation of this improvement, the increase in test values is in line with the reasoning that removing a source of between subject variability of no interest (i.e., between subject variability in overall RTs) increases sensitivity for detecting an effect of interest (i.e., an RT difference between experimental conditions).

**Figure 2:**
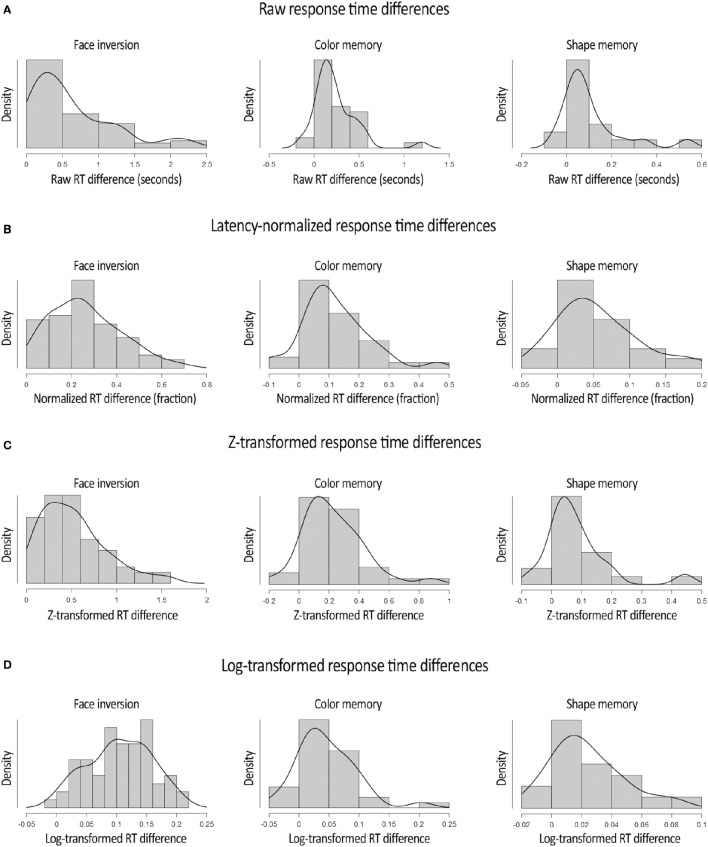
**Distributions of the raw (A)**, latency-normalized **(B)**, z-transformed **(C)**, and Log_10_-transformed **(D)**, difference in RTs between experimental conditions in all three experiments. These graphs reveal that the latency-normalization procedure had the largest impact on the RT differences of those participants that showed extremely large differences in RTs between conditions. In addition, these graphs demonstrate that after the normalization procedure, the log-transformation and (to a lesser extent) the z-transformation, the distribution of RT differences more closely followed a normal distribution. Plots were adapted from JASP Team (2016) output.

A second major asset of normalizing the RT differences in b-CFS experiments pertains to the normality of the distribution of RT differences. Indeed, the difference in raw RTs between experimental conditions tends to be skewed toward the tail end of the RT distribution (Figure 2A). For instance, in the Color experiment data, the assumption of normality is violated, according to the Shapiro-Wilk test, *W* = 0.848, *p* < 0.001.^1^ After the normalization procedure described above, however, this is no longer the case, *W* = 0.954, *p* = 0.155 (Figure 2B). Similarly, in the Shape experiment, the distribution of raw RT differences violated the assumption of normality, *W* = 0.774, *p* < 0.001, whereas the distribution of normalized RT differences did not, *W* = 0.951, *p* = 0.406. Again, in the Face Inversion experiment, the distribution of raw RT differences violated the assumption of normality *W* = 0.857, *p* < 0.001, whereas this was not (or much less) the case with the distribution of normalized RT differences, *W* = 0.966, *p* = 0.067. The distributions of the RT differences after the normalization procedure are depicted in Figure 2B. The reduction in the rightward tail-end of the RT difference by the latency-normalization procedure implies that this procedure reduces the amount of Type II errors (i.e., failing to detect an existing difference between conditions). The fact that the latency-normalized data more closely followed a normal distribution also implies greater control of Type I error rate compared to the skewed distribution of raw RT differences. Taken together, the latency-based normalization procedure offers an improved way to conduct parametric tests on b-CFS data. In addition, it reveals that the proportional (normalized) modulation of RTs in the b-CFS paradigm is approximately normally distributed (at least for the experimental manipulations provided here).

### A comparison with other normalization procedures

In order to evaluate the latency-normalization method, we also carried out two other transformations on our data sets that are widely applied to within-subject RT data: log-transformation and z-transformation (for a review, see Bush et al., 1993). Specifically, we aimed to investigate whether, within the b-CFS paradigm, RT differences between conditions would (1) yield larger effect sizes, and (2) more closely approximate a normal distribution, following these transformations as well. Z-transformations were obtained by dividing the raw RT difference between two conditions A and B (e.g., upright and inverted faces), by the standard deviation of the RT difference between these two conditions, within each participant:

$$\begin{array}{l} \Delta \text{R}{\text{T}}_{\text{Z-TRANSFORMED}}=\frac{RT_{A}-RT_{B}}{SD_{A-B}} \end{array}$$

The within-participant standard deviation for the difference in RTs between two conditions (SDA-B) was computed as follows:

$$\begin{array}{l} SD_{A-B}\;=\sqrt{\frac{\left(N_{A}-1\right)*\;SD_{A}^{2}\;+\;\left(N_{B}-1\right)*\;SD_{B}^{2}}{\left(N_{A}-1\right)+\;\left(N_{B}-1\right)}} \end{array}$$

where N_*A*_ is the number of trials in condition A, and *SD*_*A*_ is the standard deviation of the RTs in condition A. The log-transformed RT difference was obtained by first taking the logarithm with base ten of all RTs, and then computing the difference between the average^2^ log-transformed RTs of the two conditions:

$$\begin{array}{l} \Delta \text{R}{\text{T}}_{\text{LOG-TRANSFORMED}}=log_{10}\left(RT_{A}\right)-log_{10}\left(RT_{B}\right) \end{array}$$

Across our three data sets, the three transformations applied to the RT difference increased the sensitivity to detect the difference between experimental conditions (see Table 1), except for the z-transformation in the Shape experiment. Overall, although we did not compare the methods statistically, the test statistics and effect sizes for the RT difference was numerically larger after applying the log- and the latency-based normalization procedures than after applying the z-transformation procedure. In addition, the RT difference more closely approximated a normal distribution after the log-transformation and latency-normalization procedure than after the z-transformation procedure. After z-transformation, the assumption of normality was still violated in 2 out of 3 data sets. On the basis of these observations, we encourage the usage of either the latency-based normalization procedure or log-transformations to b-CFS data before conducting statistical analyses, to increase sensitivity by reducing type II error-rate.

## Results II: a monocular control condition

### Within-condition RT variability

The results of the correlation analyses between the within-condition variability in RTs (i.e., SD_WITHIN_) and the raw RT difference between experimental conditions is depicted in Figure 1B. These analyses revealed significant correlations between the within-condition variability in RTs and the raw RT difference between experimental conditions in the Face Experiment, *R* = 0.693, *p* < 0.001, BF_+0_ = 1^*^10^8^, in the Color Experiment, *R* = 0.465, *p* = 0.005, BF_+0_ = 9, and in the Shape Experiment, *R* = 0.625, *p* = 0.004, BF_+0_ = 13. Given that larger within-condition variability in RTs implies greater uncertainty, it is tentative to conclude that perceptual uncertainty positively correlates with the propensity of an experimental manipulation to affect RTs in a b-CFS paradigm. Arguably, the z-transformation described above increased effect sizes for RT differences by removing this source of between-subject variability from the RT difference between experimental conditions. We advocate caution in considering this interpretation, however, as it might be a spurious correlation emerging from the correlation between overall RTs and RT variability (e.g., Wagenmakers and Brown, 2007), which we also find in the Face Experiment, *R* = 0.797, *p* < 0.001, BF_10_ = 4^*^10^23^, in the Color Experiment, *R* = 0.736, *p* < 0.001, BF_10_ = 4^*^10^4^, and in the Shape Experiment, *R* = 0.659, *p* = 0.002, BF_10_ = 23.

### Sources of RT variability

It should be made explicit that we do not know what part of the between-subject variability in overall (i.e., median) RTs stems from individual differences in suppression durations, and what part stems from individual differences in response speed after the interocular conflict is resolved. Similarly, we do not know what part of the within-subject variability in RTs (i.e., the within-condition SD) stems from trial-to-trial differences in suppression durations, and what part stems from trial-to-trial differences in response speed after the interocular conflict is resolved. Finally, it is debated whether it is possible to unequivocally separate the contribution of these two processes that constitute b-CFS RTs (e.g., Stein et al., 2011a; Stein and Sterzer, 2014).

In order to separate the two, we capitalized on the fact that, in the Face Inversion experiment, the same participants also performed a control condition. In b-CFS control conditions, similar stimuli as in the CFS condition are used but no interocular suppression is induced. Hence, RTs in these control conditions offer insights into processes that take place after the interocular conflict is resolved. In these control conditions, both the CFS masks and the target stimuli are presented to both eyes (binocular control condition), or the CFS mask is presented to the same eye as the target stimulus, with the target stimulus displayed on top of the mask (monocular control condition). However, these control conditions typically yield null results, even when comparing stimuli that are well known to yield detection differences in other non-CFS detection paradigms, such as upright vs. inverted faces. The present Face Inversion experiment therefore adopted an improved control condition, in which face targets were blended into phase-scrambled face images (i.e., phase-scrambled noise, see Appendix for details). Face targets and phase-scrambled noise were presented to one eye, while the other eye was presented with the gray background only, such that no interocular suppression was induced.

In the Face Inversion experiment, the advantage for upright faces over inverted faces was also observed in the control condition but was smaller (9 vs. 26%) than in the CFS condition, *t*_(64)_ = 8.999, *p* < 0.001, BF_10_ = 3^*^10^10^. Overall RTs, in contrast, did not significantly differ between the CFS condition and the control condition, *t*_(64)_ = 1.506, *p* = 0.137, BF_01_ = 3. These findings, summarized in Table 2, show that the upright face advantage is more pronounced in the CFS condition than in the control condition, even though overall RTs were successfully matched between the two (for a discussion on why this is important, see Stein et al., 2011a). Taken together, upright faces are detected faster than inverted faces when there is no interocular suppression (e.g., after the interocular competition is resolved) but, the larger RT difference in the CFS condition suggests that, on top of this, upright faces are also released from interocular suppression faster than inverted faces.

**Table 2:** **General information, and effect size and normality comparisons for the face inversion control**.

**Manipulation**	**Latency/variance (SD)**	**RT difference**	**Effect (SD)**	***t*-value**	**Cohen's *d***	**BF_10_**	**W**
Control		Raw	172 ms (56)	24.606	3.052	1 × 10^31^	0.746
*N* = 65	RT_OVERALL_: 1926 ms (171)	Normalized	9% (3)	26.577	3.296	8 × 10^32^	0.938
	SD_WITHIN_: 261 ms (115)	Z-transformed	0.33 (0.11)	24.658	3.058	1 × 10^31^	0.990
		Log-transformed	0.04 (0.01)	30.157	3.741	1^*^10^36^	0.994

* *Significant violation of normality according to the Shapiro-Wilk normality test (none observed)*.

**Figure 3:**
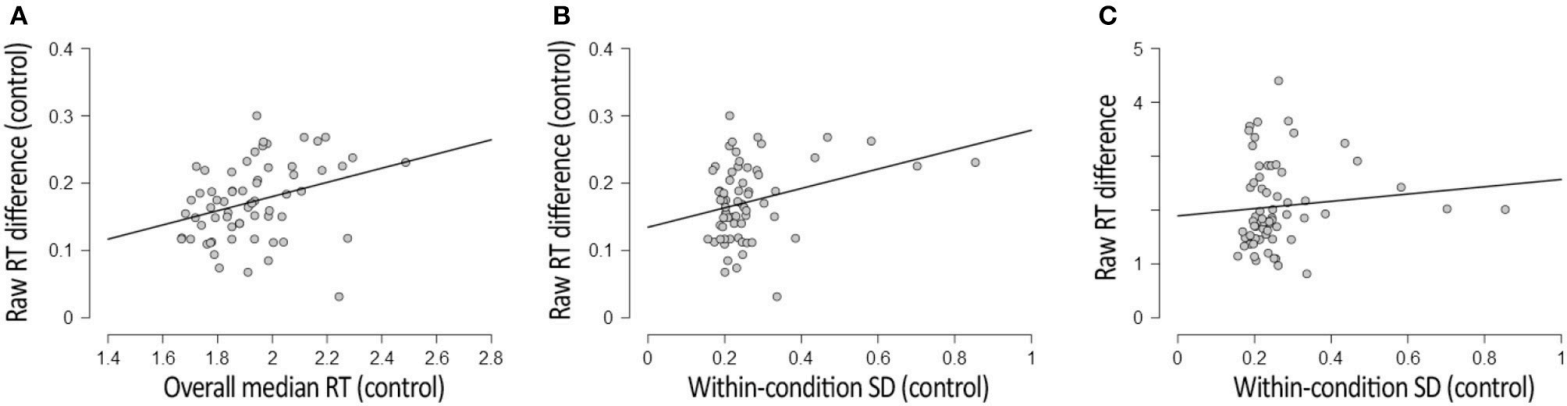
**Results of three correlational analyses involving the control condition included in the Face Inversion experiment. (A)** Depicts the correlation between participants' overall RT (x-axis) and the RT difference between upright and inverted faces (y-axis) in the control condition. **(B)** Depicts the correlation between the within-condition variability of RTs (x-axis) and the RT difference between upright and inverted faces (y-axis) in the control condition. **(C)** Depicts the (absence of a) correlation between the within-condition variability of RTs in the control condition (x-axis), and the RT difference between upright and inverted faces in the CFS condition (y-axis). Plots were adapted from JASP Team (2016) output.

Similar to the CFS condition, the RT difference between upright and inverted faces in the control condition correlated positively with the overall response speed, *R* = 0.320, *p* = 0.009, BF_+0_ = 9, and with the within-condition SD, *R* = 0.306, *p* = 0.013, BF_+0_ = 6. These correlations (depicted in Figures 3A,B respectively) were less reliable and smaller in magnitude, however, than those observed in the CFS condition.

The within-condition SD (reflecting trial-to-trial variability) was much smaller in the control condition (*M* = 292 ms, SD = 143) than in the CFS condition (*M* = 1,298 ms, SD = 835), *t*_(64)_ = 10.373, *p* < 0.001, BF_10_ = 8^*^10^13^. This observation is not surprising, considering that, in the CFS condition, our metric of within-condition variability captured both variability in suppression duration and variability in response speed after resolution of the interocular conflict, whereas in the control condition (variability in) suppression durations did not contribute to within-condition variability. The large difference in magnitude between the within-condition variability observed with and without interocular competition, however, indicates that the within-condition SD observed in the CFS condition mostly reflected trial-to-trial variability in suppression durations. In line with this view, the raw difference in RT between upright and inverted faces in the CFS condition did not correlate with the within-condition variability in the control condition, *R* = 0.016, *p* = 0.902, BF_01_ = 6 (see Figure 3C). This observation provides additional grounds for the notion that the relation between RT differences and trial-to-trial variability in RTs in the CFS condition (reported in the previous section) is primarily driven by trial-to-trial variability in suppression durations.

## General discussion

### Brief summary

Using three representative b-CFS datasets, we established that longer RTs relate to larger RTs differences between experimental conditions in the b-CFS paradigm. This may be considered a nuisance, because part of the between-subject variance that will affect statistical testing for a difference between experimental conditions (i.e., the effect of interest), does not reflect individual differences in the effect of interest, but rather reflects individual differences in overall RTs. Usually, these individual differences in overall RTs that are not specific to the experimental conditions, are of no interest to the research question. This relation between overall RTs and RT differences is probably inherent to RT data, and therefore not specific to the b-CFS paradigm (i.e., it might be a general law, following from the increase in RT variability that accompanies an increase in RT; Wagenmakers and Brown, 2007). It is of particular importance in the b-CFS paradigm, however, for two main reasons. First, in the b-CFS paradigm, overall RTs reflect both (1) the duration of interocular suppression and (2) the response speed to the stimulus after the interocular conflict is resolved. Both processes could independently elicit longer overall RT latencies and thus larger RT differences between experimental conditions than in experiments not involving CFS. Second, b-CFS is believed to be a highly potent measure to discern differences between experimental conditions because it yields large raw RT differences between conditions, with relatively few trials (e.g., compare the face inversion effect of Stein et al., 2011a, using b-CFS, with that observed in traditional detection paradigms reviewed in Lewis and Edmonds, 2003). At the same time, however, artificially stretching out RT differences between conditions, for example by presenting target stimuli at low contrast or by increasing depth of suppression through high mask contrast, also causes noise to be stretched out, allowing for more Type I errors (i.e., false positives) of a larger magnitude. For these reasons, it should become standard procedure in b-CFS experiments that examine differences between experimental conditions (the effect of interest) to investigate whether the effect of interest correlates with participants' mean RTs, and remove this between-subject variability of non-interest from the effect of interest.

### A simple solution

We encourage the usage of a simple normalization method (for similar approaches, see Tsuchiya et al., 2009; Stein et al., 2011b, 2012; Stein, 2012; Gayet et al., 2016a,b) to remove the influence of participants' overall RTs from the effect of interest. Using this method provides three clear benefits. First, removal of uninteresting between-subject variability provides a more precise measure of the effect of interest. Indeed, across all three data sets, test-statistics and effect sizes were improved after applying this normalization method to the RT differences. Second, the distribution of normalized RT difference approximates normality to a greater extent than the distribution of raw RT differences. This allows for conducting parametric tests, by transforming the data in a way that is tailored to the specifics of a participant's RT distribution, rather than being a generic approximation (e.g., such as with log-transformations). Third, normalized RT differences provide an intuitive measure of how an experimental manipulation affected participants' behavior, in the form of a proportional difference (reduction) in RTs caused by the experimental manipulation. This is an advantage over, for instance, log-transformed or z-transformed RT differences.

Log-transformed RT differences yielded increases in effect size, and approximations of normality, that were comparable to that of normalized RT differences. Therefore, log-transformations are an equally good solution for dealing with non-normal RT data with large between subject variability in the b-CFS paradigm. Z-transformations, on the other hand, were predominantly less successful in generating normally distributed RT differences.

### Suppression durations

As we argued above, RTs in a b-CFS paradigm comprise both the duration of interocular suppression, and the response speed to the stimulus after the interocular conflict is resolved. Similarly, the individual differences in (trial-to-trial variability in) RTs that we analyzed in the present study reflect variability in suppression durations, as well as variability in response speed after the interocular conflict is resolved. We suggest that the relation between individual differences in RTs and individual differences in the effect of interest, which we report here, are largely specific to CFS, reflecting individual differences in suppression durations. This notion is supported by the finding that RT differences in conditions that include interocular suppression (the CFS condition) do not correlate with individual differences in RTs drawn from conditions that do not include interocular suppression (i.e., a control condition). Another argument stems from the magnitude of the effects of interest and the magnitude of individual differences in RTs (i.e., in interocular suppression conditions). These are much larger than those typically observed in non-suppression conditions, suggesting that suppression durations constitute a large portion of the eventual RTs and RT differences. Taken together, these observations suggest that individual differences in overall RTs primarily reflect individual differences in suppression durations.

### The role of response bias

In this study, we observed that trial-to-trial variability was compellingly larger in the b-CFS suppression condition compared to the b-CFS control condition. This is potentially problematic, as the absence (or reduction) of an experimental effect in b-CFS control conditions is typically interpreted as evidence that an experimental effect in the CFS condition was not caused by anything happening *after* the interocular conflict was resolved, and thus reflects a difference in suppression duration. The difference in trial-to-trial variability between suppression and control conditions, however, could be associated with differences in perceptual uncertainty, such that response criteria might differ between suppression and control conditions. From this perspective, even the absence of an effect in a b-CFS control condition, does not necessarily preclude that an effect of interest in the b-CFS suppression condition was caused by a difference in response criteria. For example, more familiar stimuli, such as upright faces, might be more readily responded to in a state of perceptual uncertainty than less familiar stimuli, such as inverted faces (Stein et al., 2011a). In the control conditions, however, the lower levels of uncertainty could still be insufficient for the different response criteria between these conditions to modulate RTs. Future b-CFS studies could rule out the potential influence of such response biases by using non-speeded, signal detection-based bias-free protocols in which presentation times are fixed (e.g., Kaunitz et al., 2013; Lupyan and Ward, 2013; Hedger et al., 2015).

### The role of perceptual transitions

In b-CFS, an initially fully invisible stimulus eventually becomes fully visible. However, this emergence into conscious awareness is not an abrupt, all-or-none phenomenon, but a gradual one (Stein et al., 2011a), and some stimulus properties become available to consciousness earlier than others. For example, recent observations have shown that, under conditions of prolonged CFS, periods of partial awareness might arise, in which some stimulus properties (such as color) are available to consciousness, whereas other are not (such as orientations; Zadbood et al., 2011; Yang and Blake, 2012). Thus, prolonged periods of continuous flash suppression might enable distinct stages of suppression strength that vary in the degree of susceptibility to experimental manipulations. This is in line with the idea that interocular competition is modulated at different levels throughout the visual processing hierarchy (Blake and Logothetis, 2002). A recent study showed that stimuli under CFS elicited high-level behavioral priming effects only under conditions of partial awareness, but not when fully suppressed (Gelbard-Sagiv et al., 2016). Similarly, a recent study demonstrated that manipulations of attention only affected dominance durations in a binocular rivalry paradigm around the time of perceptual transitions (Dieter et al., 2015). Another binocular rivalry study showed that the detection performance of monocular probes followed gradual changes in consciousness, rather than being dichotomous (Alais et al., 2010). Thus, variations in suppression strength, as are likely to occur over the course of a b-CFS (or binocular rivalry) trial, might enable different processes to modulate suppression durations of initially fully suppressed visual input.

Both fluctuations in suppression strength during individual trials, as well as the duration of perceptual transitions may vary between observers. It is thus conceivable that our current metrics (the trial-to-trial variability in RTs, and the overall RTs) relate to the duration of transitory percepts—with limited depth of suppression—in individual participants. Accordingly, certain experimental manipulations should exert more influence on the RTs of participants with longer transitory percepts (or longer periods of shallow suppression) than on the RTs of participants with shorter transitory percepts (or shorter periods of shallow suppression). Thus, longer periods of shallow suppression may allow for a greater extent of non-conscious processing, such that larger differences in detection times can be expected for participants with longer periods of shallow suppression. The correlation between longer overall RTs (and larger trial-to-trial variability) and the RT difference between experimental conditions observed in this study fits well with this idea.

From a neural perspective, different levels of suppression might allow for neural responses to non-conscious stimuli to travel up to different levels of the (visual) processing hierarchy, thereby enabling more cognitive functions (e.g., Grill-Spector et al., 2000; Bar et al., 2001; Kanwisher, 2001; Supèr et al., 2001; Sergent and Dehaene, 2004). This leads to the expectation that, with manipulations requiring increasing cognitive demands, the relation between our current metrics and the modulation of RTs by the experimental manipulations should become more pronounced. The general consensus was, traditionally, that interocularly suppressed information cannot be processed at a semantic or conceptual level (e.g., Zimba and Blake, 1983; for a review, see Lin and He, 2009). This view stemmed from experiments using such variants of interocular suppression as binocular rivalry (Wheatstone, 1838; Alais and Blake, 2005) and flash suppression (Wolfe, 1984). In contrast, b-CFS studies have yielded contradictory findings, with some studies demonstrating that semantic or conceptual information can drive conscious access of initially suppressed visual input (e.g., Mudrik et al., 2011; Yang and Yeh, 2011; Sklar et al., 2012) while other studies have challenged this view (e.g., Heyman and Moors, 2014; Moors et al., 2016; Rabovsky et al., 2016; Stein et al., in press). Investigating the point in time (or the suppressive strength) at which a manipulation impacts conscious access in a b-CFS paradigm might be a valuable tool to resolve this apparent conflict.

## Ethics statement

All data analyzed in this study was retrieved from previously published or unpublished studies. Hence, despite human participant data was used in this study, it was already collected before this study was initiated. Hence, ethical statements are to be found in the articles from which the data was retrieved.

## Author contributions

The study idea was developed by SG and worked out in more detail by SG and TS. Data collection and analyses were conducted by SG and TS. The manuscript was initially written by SG, and was later improved by comments and suggestions provided by TS. All authors approved the final version of the manuscript.

### Conflict of interest statement

The authors declare that the research was conducted in the absence of any commercial or financial relationships that could be construed as a potential conflict of interest.

## Footnotes

^1^Individually, none of the three experiments of Gayet et al. (2013), which together constitute the data of the Color Experiment described here, violated the assumption of sphericity (all *p*'s > 0.2). As such, the usage of parametric tests for these three separate experiments was allowed, although arguably suboptimal in retrospect.

^2^In the Face experiment, the CFS masks were ramped down to zero contrast over the course of a trial, ensuring that observers would eventually perceive the target on each trial, allowing them to provide a response within the time limit. The color experiment had a relatively long time limit of ten seconds, so that targets were responded to within the time limit in virtually all trials in this experiment as well. Hence, for these two experiments a mean log-RT could be computed for each condition. In the Shape experiment, however, participants failed to respond within the time limit on an average of 19 trials (*SD* = 18) out of 144. These trials, which yielded ‘infinite’ suppression durations, could only be included in the analyses by using medians as an averaging metric (for an elaborate discussion on this approach, see Gayet et al., 2016b, Section 2.5).
