# Supplementary material for: Between-Subject Variability in the Breaking Continuous Flash Suppression Paradigm: Potential Causes, Consequences, and Solutions
Source: Front Psychol. 2017 Mar 27;8:437. doi: 10.3389/fpsyg.2017.00437 (PMC5366331; doi:10.3389/fpsyg.2017.00437)
Supplement: Supplementary file 1 [file SupplementalMethods.docx]

# Appendix: face inversion experiment methods

### Subjects

Sixty-five subjects (47 female, mean age 23.4 years, SD = 4.7) who were recruited through the University of Trento subject pool participated in the Face Inversion experiment. All participants reported normal or corrected-to-normal vision. With the exception of one participant who was also involved in conducting the experiment (as a student’s research project) all participants were naïve as to the purpose of the experiment, and received a monetary compensation for their participation.

### Stimuli

Observers viewed a 21-in. Mitsubishi CRT monitor (1024 x 768 pixels resolution, 160 Hz refresh rate) dichoptically through a custom-built mirror stereoscope. Visual stimuli were presented with Matlab (The MathWorks, Natick, MA), using the Psychtoolbox (Brainard, 1997) functions. The observer’s head was stabilized by a chin-and-head rest at a viewing distance of approximately 57 cm. The mirrors of the stereoscope were adjusted for each observer to yield stable binocular fusion. The screen was mid-gray. Throughout the experiment, two fusion contours (10.1° x 10.1°) consisting of random black and white pixels (width 0.3°) were displayed side-by-side on the screen such that one contour was shown to each eye (distance between the centers of the two contours 19.8°). In the center of each contour a small white fixation cross was presented. Participants were asked to maintain fixation throughout the experiment (moving the eyes between trials if necessary).

Target stimuli were the same grayscale face stimuli as those used by Stewart Alina, Getov, Bahrami, Todorov, and Rees (2012) and Getov, Kanai, Bahrami, and Rees (2015) (kindly provided by Spas Getov). These stimuli are computer-generated faces (using Facegen Modeller, Singular Inversions, Toronto, Canada) varying on two parameters corresponding to the facial trait dimensions of trustworthiness and dominance (see Oosterhof & Todorov, 2008, for details). The same face identity displayed three different levels of trustworthiness and dominance (-3, 0, +3, corresponding to standard deviations in the face trait dimension model), yielding a set of nine face targets (2.2° x 3.6°). These trait dimensions were not further considered for the present study. For phase scrambling and for stimulus positioning during the actual experiment face stimuli were centered within a square target image (4.0° x 4.0°) of the same mid-gray background as the rest of the screen. Target images were presented either upright or inverted (i.e., rotated by 180 degrees).

For the CFS condition, we generated 160 Mondrian-like CFS masks (9.5° x 9.5°) consisting of randomly arranged circles (diameter 0.3–1.5°). For the control condition, for every face exemplar we created 100 corresponding noise backgrounds. These noise backgrounds (9.5° x 9.5°) consisted of six phase-scrambled versions of the respective square target image that were spatially concatenated and cut to fit into the fusion contour.

### Design and procedure

The CFS and the control condition were run in separate blocks, with the control block always following the CFS block. Figure S1 shows schematic example trials from both conditions. Each trial started with a 1-s fixation period in which only the fusion contours and the fixation crosses were presented. In the CFS condition, CFS masks changing at 10 Hz were then presented to one eye, and an upright or inverted face was gradually introduced to the other eye by decreasing its transparency to zero over the first second of a trial. Beginning one second after trial onset, the contrast of the CFS masks was linearly decreased to zero over 14 s. The face was presented until response, or for a maximum trial length of 16 s. Face targets were presented in four different positions on the horizontal or vertical meridian of the fusion contours, i.e. either above, below, left or right of the fixation cross (distance of the center of the square target image to the fixation cross 2.6°, i.e. eccentricity 0.6°). Participants were asked to press one of the four arrow keys on the keyboard corresponding to the four possible face locations to indicate as fast and accurately as possible in which location a face or any part of a face became visible. In the control condition, noise backgrounds changing at 10 Hz were presented to one eye, and an upright or inverted faces was gradually introduced to the same eye by decreasing its transparency to zero over 3.6 s. Face locations and the location task were identical to the CFS condition. Trials with incorrect or no localization responses (CFS condition: M = 0.02%, SD 0.04; control condition: M = 0.01%, SD 0.02) were excluded from further analyses.

Both the CFS and the control condition blocks consisted of 288 trials, in which all combinations of two eyes for target presentation, two face orientations, nine face identities, and four face locations occurred twice. Trial order was randomized. The experimental blocks were separated by two obligatory breaks after 96 and 192 trials, respectively. Before starting the experimental blocks, participants received at least 12 practice trials.


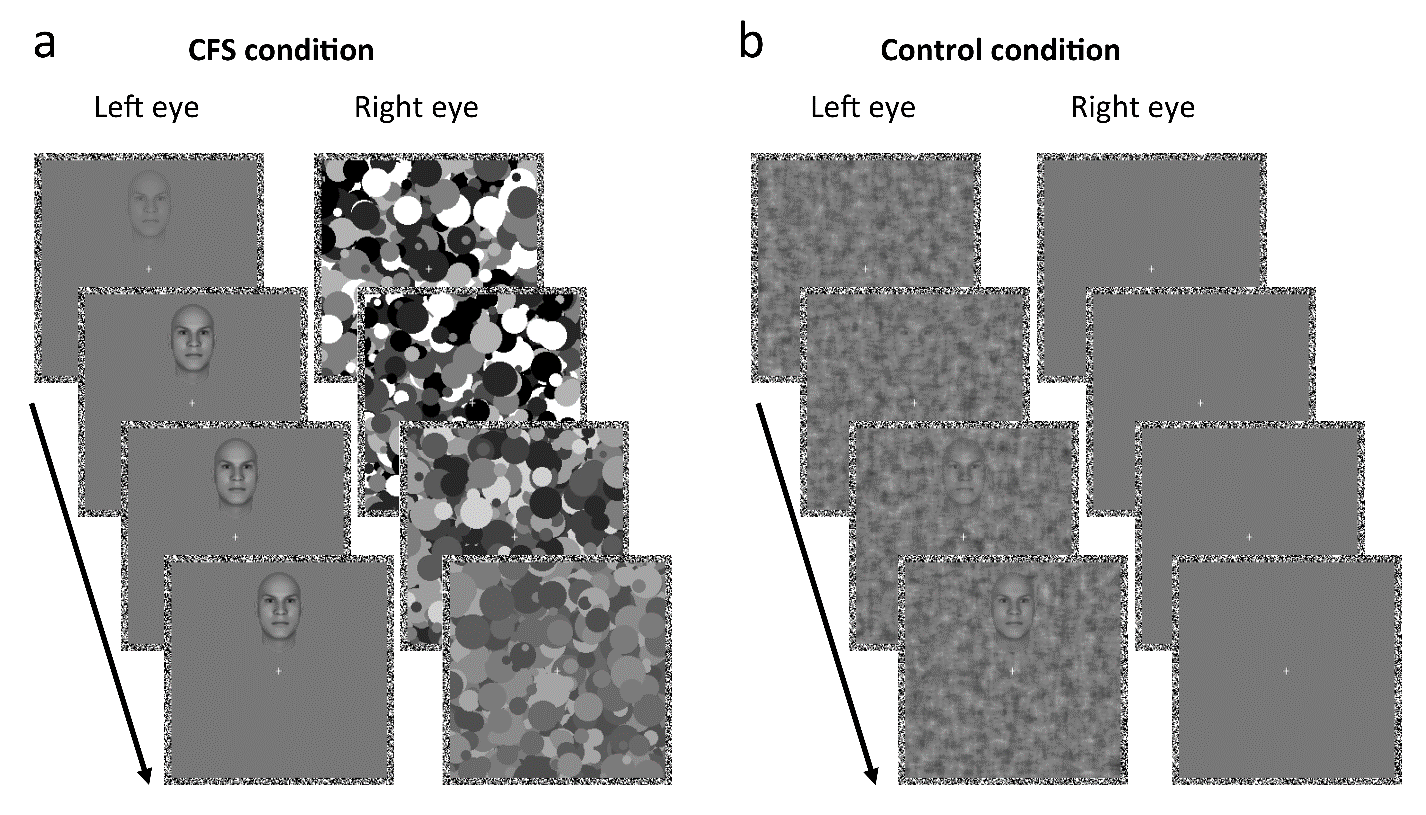


**Figure S1.** Schematic trials of the (a) CFS condition and (b) control condition from the Face Inversion experiment. In the CFS condition, a face target was presented to one eye and Mondrian-like CFS masks updated at 10 Hz to the other eye. In the control condition, the face target was presented to the same eye as the phase-scrambled noise. No stimulus was presented to the other eye, thus no interocular suppression was induced.

### Additional references

Stewart, L. H., Ajina, S., Getov, S., Bahrami, B., Todorov, A., & Rees, G. (2012). Unconscious evaluation of faces on social dimensions. *Journal of Experimental Psychology: General*, *141*(4), 715-727.

Getov, S., Kanai, R., Bahrami, B., & Rees, G. (2015). Human brain structure predicts individual differences in preconscious evaluation of facial dominance and trustworthiness. *Social cognitive and affective neuroscience*, *10*(5), 690-699.
